# *ADCY5*, *CAPN10* and *JAZF1* Gene Polymorphisms and Placental Expression in Women with Gestational Diabetes

**DOI:** 10.3390/life11080806

**Published:** 2021-08-09

**Authors:** Przemysław Ustianowski, Damian Malinowski, Patrycja Kopytko, Michał Czerewaty, Maciej Tarnowski, Violetta Dziedziejko, Krzysztof Safranow, Andrzej Pawlik

**Affiliations:** 1 Department of Obstetrics and Gynecology, Pomeranian Medical University, 70-111 Szczecin, Poland; przemyslaw.ustianowski@pum.edu.pl; 2 Department of Experimental and Clinical Pharmacology, Pomeranian Medical University, 70-111 Szczecin, Poland; damian.malinowski@pum.edu.pl; 3 Department of Physiology, Pomeranian Medical University, 70-111 Szczecin, Poland; patrycja.kopytko@op.pl (P.K.); michal.czerewaty@wp.pl (M.C.); maciejt@pum.edu.pl (M.T.); 4 Department of Biochemistry and Medical Chemistry, Pomeranian Medical University, 70-111 Szczecin, Poland; viola@pum.edu.pl (V.D.); chrissaf@mp.pl (K.S.)

**Keywords:** gestational diabetes, polymorphism, placenta

## Abstract

Gestational diabetes mellitus (GDM) is carbohydrate intolerance that occurs during pregnancy. This disease may lead to various maternal and neonatal complications; therefore, early diagnosis is very important. Because of the similarity in pathogenesis of type 2 diabetes and GDM, the genetic variants associated with type 2 diabetes are commonly investigated in GDM. The aim of the present study was to examine the associations between the polymorphisms in the *ADCY5* (rs11708067, rs2877716), *CAPN10* (rs2975760, rs3792267), and *JAZF1* (rs864745) genes and GDM as well as to determine the expression of these genes in the placenta. This study included 272 pregnant women with GDM and 348 pregnant women with normal glucose tolerance. The diagnosis of GDM was based on a 75 g oral glucose tolerance test (OGTT) at 24–28 weeks gestation, according to International Association of Diabetes and Pregnancy Study Groups (IADPSG) criteria. There were no statistically significant differences in the distribution of the *ADCY5* gene (rs11708067, rs2877716) and *CAPN10* gene (rs2975760, rs3792267) polymorphisms between pregnant women with normal carbohydrate tolerance and pregnant women with GDM. We have shown a lower frequency of *JAZF1* gene rs864745 C allele carriers among women with GDM CC + CT vs. TT (OR = 0.60, 95% CI = 0.41–0.87, *p* = 0.006), and C vs. T (OR = 0.75, 95% CI = 0.60–0.95, *p* = 0.014). In addition, *ADCY5* and *JAZF1* gene expression was statistically significantly increased in the placentas of women with GDM compared with that of healthy women. The expression of the *CAPN10* gene did not differ significantly between women with and without GDM. Our results indicate increased expression of *JAZF1* and *ADCY5* genes in the placentas of women with GDM as well as a protective effect of the C allele of the *JAZF1* rs864745 gene polymorphism on the development of GDM in pregnant women.

## 1. Introduction

Gestational diabetes mellitus (GDM) is the metabolic disorder occurring during pregnancy. GDM may lead to various maternal and neonatal complications; therefore, early diagnosis and therapy of this disease are very important [1]. The factors predisposing to GDM development are commonly searched. Known risk factors for GDM include maternal age, obesity, impaired carbohydrate tolerance before pregnancy, and history of GDM during previous pregnancies [2,3]. In recent years, many genetic and non-genetic factors that may predispose women to GDM have been sought. Various genetic polymorphisms are being studied, as well as the expression of genes in human umbilical vein endothelial cells and in the placenta [4,5]. There are many similarities in the pathogenesis of GDM and type 2 diabetes [6]; therefore, genetic polymorphisms associated with type 2 diabetes are taken into account. Previous studies have shown that polymorphisms of *ADCY5*, *CAPN10,* and *JAZF1* genes may be associated with an increased risk of type 2 diabetes [7,8,9]. 

*ADCY5* encodes adenylate cyclase 5, which catalyzes the generation of cyclic AMP, which regulates insulin secretion in the pancreatic islet β-cell [10]. Moreover, *ADCY5* may be involved in carbohydrate metabolism and glucose-stimulated insulin secretion [11,12,13]. 

*CAPN10* gene encodes calpain-10, a protein that is a cysteine protease. The expression of the *CAPN10* gene, located in 2q37.3, was detected in various tissues; however, mainly in the liver, skeletal muscle, and pancreatic islet β cells [14]. *CAPN10* gene takes part in cell apoptosis, proliferation, and differentiation processes. *CAPN10* gene regulates intracellular signal transduction, adipocyte differentiation, and insulin secretion [15]. 

Juxtaposed with another zinc finger gene 1 (*JAZF1*) encodes a putative transcription factor that interacts with protein NR2C2 (nuclear receptor subfamily 2, group C, member 2) and regulates the expression of many genes involved in carbohydrate and lipid metabolism [16]. 

The aim of the present study was to examine the associations between the polymorphisms of the *ADCY5* gene (rs11708067, rs2877716), *CAPN10* gene (rs2975760, rs3792267), and *JAZF1* gene rs864745 and GDM as well as to determine the expression of these genes in the placenta. 

## 2. Materials and Methods

### 2.1. Participants

This case-control study included 272 pregnant women with GDM and 348 pregnant women with normal glucose tolerance (NGT). GDM was diagnosed on the basis of a 75 g oral glucose tolerance test (OGTT) at 24–28 weeks gestation, according to the International Association of Diabetes and Pregnancy Study Groups (IADPSG) criteria [17]. The diagnosis of GDM was made when one of the following plasma glucose values in the OGTT was met or exceeded: fasting plasma glucose of 92 mg/dL (5.1 mmol/L), 1 h plasma glucose of 180 mg/dL (10.0 mmol/L), or 2 h plasma glucose of 153 mg/dL (8.5 mmol/L). Among pregnant women with GDM, 78% of women were treated with diet alone throughout pregnancy, and the remaining 22% used diet and insulin until delivery. The exclusion criteria were acute or chronic complications, such as diabetic ketoacidosis, or other disorders affecting glucose metabolism, chronic inflammatory diseases, and autoimmune diseases. The study was approved by the Ethics Committee of Pomeranian Medical University, Szczecin, Poland (KB-0012/40/14), and written informed consent was obtained from all subjects.

### 2.2. Methods

All samples were genotyped in duplicate using allelic discrimination assays with TaqMan^®^ probes (Applied Biosystems, Carlsbad, CA, USA) on a 7500 Fast Real-Time PCR Detection System (Applied Biosystems, Carlsbad, CA, USA). In order to discriminate the polymorphisms, we employed TaqMan^®^ Pre-Designed SNP Genotyping Assays, including appropriate primers and fluorescently labelled (FAM and VIC) MGB™ probes to detect the alleles.

### 2.3. RNA Isolation

Human placentas were obtained from the Department of Obstetrics and Gynecology, Pomeranian Medical University, from 77 women (34 healthy women and 43 with GDM) within 10 min of delivery. Each placenta was then weighed and sampled in placental explants (~100 mg wet weight) from the maternal villous placenta region. Tissues were dissected to remove visible connective tissue, vessels, and calcium deposits. Explants were homogenized, and total RNA was isolated accordingly to the manufacturer’s protocol using the RNAsy Mini Kit (RNeasy^®^ Mini Kit, Qiagen, Hilden, Germany). 

### 2.4. Reverse-Transcription (RT-PCR)

Isolated messenger mRNA was reverse-transcribed using the cDNA synthesis Kit (RevertAid RT Kit, Thermo Scientific, Waltham, MA, USA) according to the manufacturer’s protocol. 

### 2.5. Real-Time Quantitative Reverse Transcription PCR (RQ-PCR)

Quantitative expression analysis of the selected genes, as well as the beta2-microglobulin reference gene, was performed using real-time RT-PCR on an ABI PRISM^®^ Fast 7500 Sequence Detection System (Applied Biosystems, Waltham, MA, USA). Real-time conditions were as follows: 95 °C (15 s), 40 cycles at 95 °C (15 s), and 60 °C (1 min). According to melting point analysis, only one PCR product was amplified under these conditions. The relative quantity of a target was normalized to the endogenous control β-2 microglobulin gene.

### 2.6. Statistical Analysis

The consistency of the genotype distribution with Hardy–Weinberg equilibrium (HWE) was assessed using the exact test. A chi-square test was used to compare the genotype and allele distributions between the groups. Quantitative variables were compared between the genotype groups using the Mann–Whitney U test. A multivariate logistic regression model was used to find independent predictors of GDM risk. *p*-values < 0.05 were considered statistically significant. 

The statistical power of the study at the 0.05 significance level with 272 GDM patients and 348 control subjects was sufficient to detect with 80% probability the real effect size of genotype–phenotype associations corresponding to odds ratio 0.60 or 1.54 for *CAPN10* rs2975760, 0.62 or 1.51 for *ADCY5* rs11708067, 0.63 or 1.49 for *ADCY5* rs2877716, 0.70 or 1.40 for *CAPN10* rs3792267, and 0.72 or 1.38 for *JAZF1* rs864745 when comparing allele frequencies between groups.

## 3. Results

The characteristics of the women included in the study are shown in Table 1. The distribution of studied genotypes is presented in Table 2. As shown, there were no statistically significant differences in the distribution of the *ADCY5* gene (rs11708067, rs2877716) and *CAPN10* gene (rs2975760, rs3792267) polymorphisms between pregnant women with normal carbohydrate tolerance and pregnant women with GDM. We found a lower frequency of the *JAZF1* gene rs864745 C allele carriers among women with GDM CC+CT vs. TT (odds ratio (OR) = 0.60, 95% confidence interval (CI) = 0.41–0.87, *p* = 0.006), and C vs. T (OR = 0.75, 95% CI = 0.60–0.95, *p* = 0.014). These associations remained significant in the logistic regression model adjusted for body mass index before pregnancy: CC+CT vs. TT (odds ratio (OR) = 0.61, 95% confidence interval (CI) = 0.41–0.91, *p* = 0.014), and C vs. T (OR = 0.75, 95% CI = 0.58–0.96, *p* = 0.021).

We also examined the associations between the studied polymorphisms and clinical parameters, such as the results of the oral fasting glucose tolerance test, daily insulin requirement, body mass before pregnancy, body mass at birth, body mass increase during pregnancy, body mass index (BMI) before pregnancy, BMI at birth, BMI increase during pregnancy, newborn body mass, and APGAR scores. We have shown increased daily insulin requirements in women with *ADCY5* gene rs11708067 AG and rs2877716 genotype CT genotypes, higher APGAR scores in newborns from women with *CAPN10* gene rs2975760 CC genotype, and higher newborn body mass from women with *CAPN10* gene rs3792267 AA genotype (Tables S1–S5, Supplementary Material).

**Figure 1:**
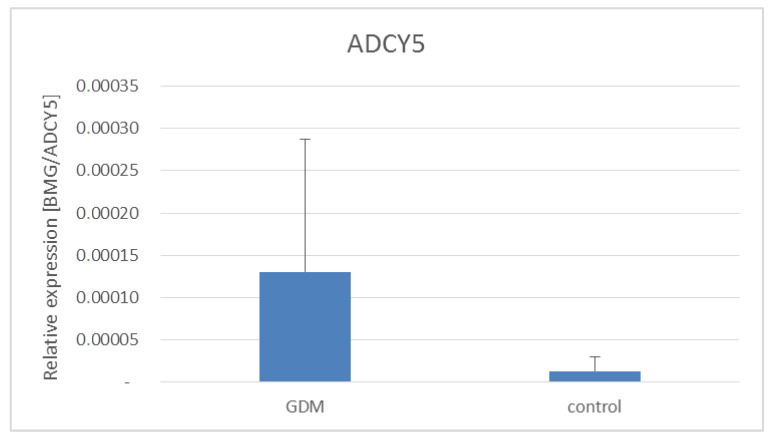
Expression of *ADYC5* gene in placentas of women with and without GDM. GDM vs. healthy women, *p* < 0.00001—Mann–Whitney U test.

**Figure 2:**
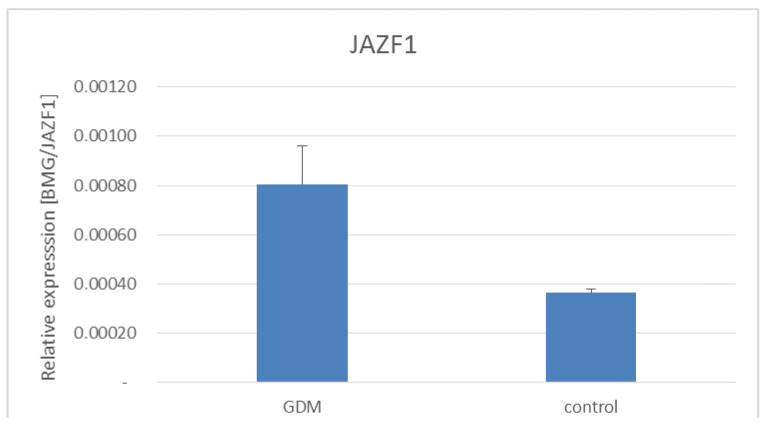
Expression of *JAZF1* gene in placentas of women with and without GDM. GDM vs. healthy women, *p* = 0.0006—Mann–Whitney U test.

**Figure 3:**
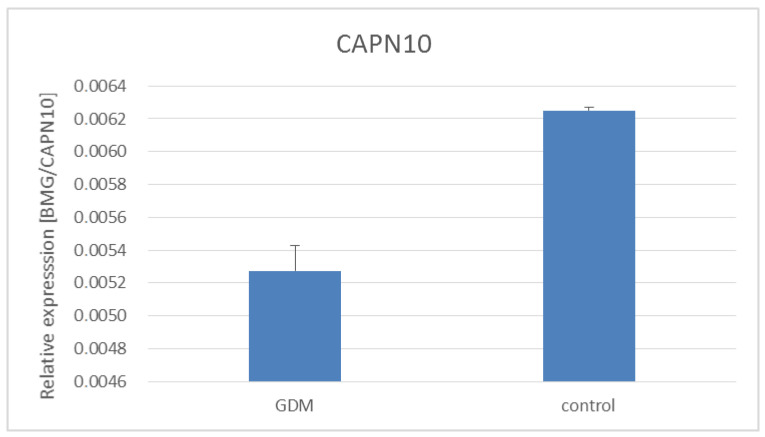
Expression of *CAPN10* gene in placentas of women with and without GDM. GDM vs. healthy women, *p* = 0.17—Mann–Whitney U test.

Additionally, we examined the expression of *ADCY5*, *CAPN10,* and *JAZF1* genes in the placentas of women with GDM and healthy women (Figure 1, Figure 2 and Figure 3). As shown in Figure 1 and Figure 2 *ADCY5* and *JAZF1* gene expression was significantly increased in women with GDM. 

We also analyzed the correlations between studied gene polymorphisms and the expression of respective genes in the placentas of women with and without GDM. Expression of the *JAZF1* gene in the placenta was significantly higher in control group women with rs864745 CC genotype when compared to CT genotype (mean ± SD: TT = 0.00032 ± 0.00054; CT = 0.00012 ± 0.00019; CC = 0.00070 ± 0.00061; CC vs. CT, *p* = 0.013 U Mann-Whitney test). No other significant associations between genotypes and placental expression of the respective genes were found in any of the groups.

## 4. Discussion

In this study, we examined the associations between the polymorphisms of the *ADCY5* gene (rs11708067, rs2877716), *CAPN10* gene (rs2975760, rs3792267), *JAZF1* gene rs864745, and GDM. We found a lack of statistically significant associations between the polymorphisms of the *ADCY5* gene (rs11708067, rs2877716), *CAPN10* gene (rs2975760 rs3792267), and GDM. We have indicated a lower frequency of *JAZF1* gene rs864745 C allele carriers among women with GDM, suggesting that this allele may protect against GDM development. Additionally, the *JAZF1* and *ADCY5* gene expression was significantly increased in women with GDM. Previous studies investigated in various populations the associations between the polymorphisms of the *ADCY5* gene (rs11708067, rs2877716), *CAPN10* gene (rs2975760, rs3792267), *JAZF1* gene rs864745, and type 2 diabetes [7,8,9]. It has been shown that these polymorphisms can exhibit functional properties by affecting transcriptional activity, mRNA, and protein expression. These functional regulatory variants were associated in GWAS with risk of type 2 diabetes and also affected insulin secretion and glycemic profile. In addition, they affect gene expression in various tissues including pancreatic islets [7,12,18,19]. Because of the similarity between the pathogenesis of type 2 diabetes and gestational diabetes, these polymorphisms were also studied in women with GDM of various populations [20]. Arora et al. have shown that the rs11708067 polymorphism of the *ADCY5* gene may protect against GDM development in North Indian women [21]. The results of the study by Andersson et al. suggest that this polymorphism may be associated with decreased newborn birthweight [22]. Khan et al. found a lack of statistically significant association between *CAPN10* gene rs2975760 polymorphism and GDM in Indian women [23]. Similarly, there were no statistically significant associations between rs2975760 and rs3792267 polymorphisms of the *CAPN10* gene and GDM in the Chinese population [24]. Stuebe et al. have shown an association between *JAZF1* gene rs864745 polymorphism and GDM among African-American women [25]. The results of our study suggest an association between *JAZF1* gene rs864745 polymorphism and GDM. The expression of the *JAZF1* gene was detected in various tissues, especially involved in tissues involved in carbohydrate metabolism, such as the pancreas, liver, skeletal muscle, and fat tissue [26]. *JAZF1* plays an important role in glucose metabolism and is involved in insulin sensitivity, insulin resistance, gluconeogenesis, lipid metabolism, and inflammatory processes [27]. It has been shown that some diseases, such as diabetes, obesity, and hepatic steatosis, may alter *JAZF1* expression in tissues [28,29,30]. Reduced *JAZF1* expression was also noted in mice on high-fat diets and patients with type 2 diabetes [31,32,33]. This may suggest that hyperglycaemia and dyslipidaemia may reduce *JAZF1* expression. It has been shown that *JAZF1* protein may modulate the expression of various genes involved in cellular metabolism, apoptosis, mitochondrial function, and oxidative stress [28]. *JAZF1* plays an important role in the regulation of the inflammatory process in fat tissue. It has been shown that mice with increased expression of *JAZF1* exhibited decreased insulin resistance and reduced inflammation in fat tissue with decreased release of pro-inflammatory cytokines [33]. *JAZF1* may prevent lipid accumulation through regulation of lipid synthesis and oxidation and has been shown to decrease lipid synthesis in both liver and adipose tissue. *JAZF1* adenovirus-treated adipocytes showed reduced triglyceride content and prolipogenic gene expression [28]. In addition, *JAZF1* has been shown to have an important function in regulating lipolytic processes. Increasing *JAZF1* expression stimulates lipolysis by increasing the expression of triglyceride lipase (ATGL) and hormone-sensitive lipase (HSL) in both the liver and adipocytes [29]. Previous studies have shown that *JAZF1* regulates carbohydrate metabolism, preventing hyperglycemia through the regulation of the enzymes phosphoenolpyruvate carboxykinase (PEPCK) and glucose-6-phosphatase [27,30]. Mice with increased *JAZF1* expression showed marked improvement in insulin resistance and metabolic profile with reduced fasting plasma insulin and glucose levels [27,30]. *JAZF1* has also been shown to affect the expression of gluconeogenic genes in the liver [34]. Moreover, *JAZF1* decreases insulin resistance by increasing the expression of glucose transporters GLUT in the liver, adipose tissue, skeletal, muscle, and cardiomyocytes [27,30]. Therefore, *JAZF1* facilitates glucose transport in various tissues, especially in liver and adipose tissue. In our study, we have shown increased expression of the *JAZF1* gene in the placentas of women with GDM. We hypothesize that increased expression of the *JAZF1* gene in the placentas of women with GDM may be one of the compensatory mechanisms protecting the fetus from the effects of maternal excessive glycaemia. 

Calpain-10 plays important role in glucose-stimulated insulin secretion [35]. It has been shown that calpain-10 inhibition decreases insulin release, and the expression of calpain-10 in pancreatic beta cells correlated positively with insulin secretion in response to glucose stimulation [36]. Diabetic patients showed decreased calpain-10 expression in the pancreas and skeletal muscle, which was associated with insulin resistance [37,38]. Moreover, pharmacologic inhibition of calpain 10 decreased the activity of glucose transporter GLUT4 in skeletal muscle [39]. However, we did not show an association between the *CAPN10* gene rs2975760, rs3792267 polymorphisms, and GDM as well as differences in *CAPN10* gene expression between women with and without GDM.

*ADCY5* is involved in cAMP generation, which regulates insulin release in the pancreatic islet beta-cells. In our study, there were no statistically significant associations between *ADCY5* gene polymorphisms and GDM; however, the expression of the *ADCY5* gene was significantly increased in women with GDM. Hodson et al. have shown that *ADCY5* gene rs11708067 polymorphisms influence the ADCY5 mRNA expression in pancreatic islets and ADCY5 is required for glucose coupling to insulin secretion in human islets [12]. Changes in ADCY5 expression in β-cells and impaired glucose signaling represent a likely pathway through which *ADCY5* gene polymorphisms affect fasting glucose levels and diabetes risk. Roman et al. have indicated that the *ADCY5* gene rs11708067A allele was associated with lower transcriptional activity and stronger binding of nuclear proteins in pancreatic islets [7].

We also examined the associations between studied polymorphisms and the expression of respective genes in the placentas of women with and without GDM. Expression of the *JAZF1* gene in the placenta was significantly higher in control group women with rs864745 CC genotype when compared to CT genotype. There were no other significant associations between genotypes and placental expression of the respective genes in any of the groups. We suppose that factors other than genetic polymorphisms may have a stronger effect on the expression of these genes in the placenta, especially in women with GDM.

The results of our study suggest an association between *JAZF1* gene rs864745 polymorphism and GDM, as well as increased expression of *JAZF1* and *ADCY5* genes in the placentas of women with GDM. Grarup et al. have shown that carriers of the *JAZF1* gene rs864745 T allele had decreased insulin release in response to glucose administration [19]. In our study, women with this T allele had a higher risk of GDM development. 

## 5. Conclusions

Our results indicate increased expression of *JAZF1* and *ADCY5* genes in the placentas of women with GDM as well as a protective effect of the C allele of the *JAZF1* rs864745 gene polymorphism on the development of GDM in pregnant women. However, this association should be confirmed in larger multicenter studies.

## Figures and Tables

**Table 1:** The characteristics of the women included in the study.

Parameters	Control Group	GDM	*p*-Value ^&^
	Mean ± SD	Mean ± SD	
Age (years)	30.3 ± 4.6	31.7 ± 5.4	0.00014
Height (cm)	166.1 ± 6.1	165.9 ± 6.2	0.75
Pregnancy number	1.9 ± 1.1	2.1 ± 1.2	0.27
Fasting glucose in an oral glucose tolerance test (mg/dL)	80.3 ± 6.7	95.7 ± 11.6	<0.00001
Glucose after 1 h in an oral glucose tolerance test (mg/dL)	122.2 ± 23.6	156.9 ± 37.1	<0.00001
Glucose after 2 h in an oral glucose tolerance test (mg/dL)	102.3 ± 18.7	130.9 ± 34.1	<0.00001
Body mass before pregnancy (kg)	64.3 ± 13.4	73.3 ± 17.5	<0.00001
Body mass at birth (kg)	78.1 ± 13.4	84.8 ± 15.9	<0.00001
Body mass increase during pregnancy (kg)	13.8 ± 5.4	11.5 ± 7.2	<0.00001
BMI before pregnancy (kg/m^2^)	23.3 ± 4.3	26.6 ± 6.2	<0.00001
BMI at birth (kg/m^2^)	28.3 ± 4.3	30.8 ± 5.7	<0.00001
BMI increase during pregnancy (kg/m^2^)	5.0 ± 1.9	4.2 ± 2.7	<0.00001
Newborn body mass (g)	3287 ± 529	3286 ± 566	0.97
APGAR (0–10)	9.4 ± 1.0	9.3 ± 1.1	0.13

^&^ Mann-Whitney U test.

**Table 2:** Distribution of *ADCY5*, *CAPN10*, and *JAZF1* genotypes and alleles in women with GDM and control group.

Genotypes and Alleles	Control Group		GDM		*p*-Value ^^^	Compared Genotypes or Alleles	OR (95% CI)	*p*-Value ^^^
	n	%	n	%				
***ADCY5*** **rs11708067 genotype**								
AA	244	70.11%	193	70.96%	0.69	GG+AG vs. AA	0.96 (0.68–1.36)	0.82
AG	97	27.87%	71	26.10%		GG vs. AG+AA	1.48 (0.53–4.12)	0.45
GG	7	2.01%	8	2.94%		GG vs. AA	1.44 (0.51–4.05)	0.48
						AG vs. AA	0.93 (0.65–1.33)	0.67
						AG vs. AA	1.56 (0.54–4.50)	0.41
**Allele**								
A	585	84.05%	457	84.01%		G vs. A	1.00 (0.74–1.36)	0.98
G	111	15.95%	87	15.99%				
***ADCY5*** **rs2877716 genotype**								
CC	237	68.10%	185	68.01%	1.00	TT+CT vs. CC	1.00 (0.71–1.41)	0.98
CT	102	29.31%	80	29.41%		TT vs. CT+CC	0.99 (0.37–2.71)	0.99
TT	9	2.59%	7	2.57%		TT vs. CC	1.00 (0.36–2.73)	0.99
						CT vs. CC	1.00 (0.71–1.43)	0.98
						TT vs. CT	0.99 (0.35–2.78)	0.99
**Allele**								
C	576	82.76%	450	82.72%		T vs. C	1.00 (0.75–1.35)	0.99
T	120	17.24%	94	17.28%				
***CAPN10*** **rs2975760 genotype**								
TT	261	75.22%	196	72.59%	0.74	CC+TC vs. TT	1.15 (0.80–1.65)	0.46
TC	77	22.19%	67	24.81%		CC vs. TC+TT	1.00 (0.37–2.72)	1.00
CC	9	2.59%	7	2.59%		CC vs. TT	1.04 (0.38–2.83)	0.95
						TC vs. TT	1.16 (0.80–1.69)	0.44
						CC vs. TC	0.89 (0.32–2.53)	0.83
**Allele**								
T	599	86.31%	459	85.00%		C vs. T	1.11 (0.81–1.53)	0.51
C	95	13.69%	81	15.00%				
***CAPN10* rs3792267 genotype**								
GG	175	50.43%	132	48.89%	0.74	AA+GA vs. GG	1.06 (0.77–1.46)	0.70
GA	129	37.18%	108	40.00%		AA vs. GA+GG	0.88 (0.54–1.45)	0.63
AA	43	12.39%	30	11.11%		AA vs. GG	0.92 (0.55–1.55)	0.77
						GA vs. GG	1.11 (0.79–1.56)	0.55
						AA vs. GA	0.83 (0.49–1.42)	0.50
**Allele**						A vs. G	1.01 (0.79–1.28)	0.96
G	479	69.02%	372	68.89%				
A	215	30.98%	168	31.11%				
***JAZF1* rs864745 genotype**								
TT	69	19.88%	79	29.26%	0.022	CC+CT vs. TT	0.60 (0.41–0.87)	0.006
CT	191	55.04%	136	50.37%		CC vs. CT+TT	0.76 (0.52–1.12)	0.17
CC	87	25.07%	55	20.37%		CC vs. TT	0.55 (0.35–0.88)	0.012
						CT vs. TT	0.62 (0.42–0.92)	0.017
						CC vs. CT	0.89 (0.59–1.33)	0.56
**Allele**								
T	329	47.41%	294	54.44%		C vs. T	0.75 (0.60–0.95)	0.014
C	365	52.59%	246	45.56%				

^^^ χ^2^ test. HWE: control group *p* = 0.55, GDM group *p* = 0.65 for *ADCY5* rs11708067. HWE: control group *p* = 0.71, GDM group *p* = 0.83 for *ADCY5* rs2877716. HWE: control group *p* = 0.26, GDM group *p* = 0.63 for *CAPN10* rs2975760. HWE: control group *p* = 0.02, GDM group *p* = 0.26 for *CAPN10* rs3792267. HWE: control group *p* = 0.07, GDM group *p* = 0.90 for *JAZF1* rs864745.

## Data Availability

Not applicable.

## Supplementary Materials

The following are available online at www.mdpi.com/article/10.3390/life11080806/s1, Table S1: Clinical parameters of women with GDM stratified according to ADCY5 rs11708067 genotype, Table S2: Clinical parameters of women with GDM stratified according to ADCY5 rs2877716 genotype, Table S3: Clinical parameters of women with GDM stratified according to CAPN10 rs2975760 genotype, Table S4: Clinical parameters of women with GDM stratified according to CAPN10 rs3792267 genotype, Table S5. Clinical parameters of women with GDM stratified according to JAZF1 rs864745 genotype.
