# Supplementary material for: ADCY5, CAPN10 and JAZF1 Gene Polymorphisms and Placental Expression in Women with Gestational Diabetes
Source: Life (Basel). 2021 Aug 9;11(8):806. doi: 10.3390/life11080806 (PMC8399092; doi:10.3390/life11080806)
Supplement: Supplementary file 1 [file life-11-00806-s001.zip › life-1299143-supplementary.pdf]

Supplementary data of:

ADCY5, CAPN10 and *JAZF1* Gene Polymorphisms and Placental Expression in Women with Gestational Diabetes

Przemysław Ustianowski [1], Damian Malinowski [2], Patrycja Kopytko [3], Michał Czerewaty [3], Maciej Tarnowski [3], Violetta Dziedziejko [4], Krzysztof Safranow [4] and Andrzej Pawlik [3,*]

[1] Department of Obstetrics and Gynecology, Pomeranian Medical University, 70-111 Szczecin, Poland; przemyslaw.ustianowski@pum.edu.pl
[2] Department of Experimental and Clinical Pharmacology, Pomeranian Medical University, 70-111 Szczecin, Poland; damian.malinowski@pum.edu.pl
[3] Department of Physiology, Pomeranian Medical University, 70-111 Szczecin, Poland; patrycja.kopytko@op.pl (P.K.); michal.czerewaty@wp.pl (M.C.); maciejt@pum.edu.pl (M.T.)
[4] Department of Biochemistry and Medical Chemistry, Pomeranian Medical University, 70-111 Szczecin, Poland; viola@pum.edu.pl (V.D.); chrissaf@mp.pl (K.S.)
* Correspondence: pawand@poczta.onet.pl

Table S1. Clinical parameters of women with GDM stratified according to *ADCY5* rs11708067 genotype.

Parameters	***ADCY5* rs11708067 Genotype**					
	**AA n=193**	**AG n=71**	**GG n=8**	**AA vs AG**	**AA vs GG**	**AG vs GG**
	**Mean ± SD**	**Mean ± SD**	**Mean ± SD**	**p[&]**		
Fasting glucose in an oral glucose tolerance test (mg/dL)	95.6±12.4	96.6±10.1	97.0±10.4	0.11	0.64	0.43
Glucose after 1 h in an oral glucose tolerance test (mg/dL)	155.4±36.2	158.9±41.2	151.1±33.1	0.62	0.89	0.72
Glucose after 2 h in an oral glucose tolerance test (mg/dL)	131.6±36.5	128.4±30.5	118.5±28.3	0.60	0.35	0.44
Daily insulin requirement [unit]	14.5±21.7	26.7±43.2	10.5±13.6	0.002	1.00	0.20
Body mass before pregnancy [kg]	72.6±16.1	75.1±18.5	76.1±21.7	0.44	0.81	0.99
Body mass at birth [kg]	84.3±14.7	86.3±17.5	87.1±18.5	0.58	0.84	0.97
Body mass increase during pregnancy [kg]	11.8±7.2	11.2±6.8	11.0±6.5	0.44	0.75	0.94
BMI before pregnancy [kg/m^2]	26.4±5.6	27.1±6.4	27.1±8.1	0.59	0.90	0.75
BMI at birth [kg/m^2]	30.7±5.2	31.2±6.2	31.0±7.2	0.81	0.91	0.91
BMI increase during pregnancy [kg/m^2]	4.3±2.6	4.1±2.5	3.9±2.4	0.37	0.62	0.90
Newborn body mass [g]	3281±566	3282±552	3093±634	0.70	0.41	0.45
APGAR [0-10]	9.2±1.1	9.4±0.9	9.5±0.9	0.60	0.39	0.50

[&] Mann-Whitney U test.

Table S2. Clinical parameters of women with GDM stratified according to *ADCY5* rs2877716 genotype.

Parameters	*ADCY5* rs2877716 Genotype					
	**CC** **n=185**	**CT** n=80	**TT** n=7	**CC vs CT**	**CC vs TT**	**CT vs TT**
	**Mean ± SD**	**Mean ± SD**	**Mean ± SD**		**p[&]**	
Fasting glucose in an oral glucose tolerance test (mg/dL)	95.5±12.7	96.4±8.2	99.9±18.7	0.08	0.96	0.84
Glucose after 1 h in an oral glucose tolerance test (mg/dL)	155.2±36.5	157.2±38.1	171.0±54.0	0.64	0.44	0.53
Glucose after 2 h in an oral glucose tolerance test (mg/dL)	132.0±37.1	125.9±28.9	140.9±34.7	0.26	0.53	0.31
Daily insulin requirement [unit]	14.7±22.0	24.2±41.1	17.4±25.6	0.015	0.80	0.57
Body mass before pregnancy [kg]	72.6±16.3	75.3±18.3	69.8±16.3	0.33	0.54	0.44
Body mass at birth [kg]	84.3±15.0	86.6±16.7	82.6±18.0	0.42	0.55	0.53
Body mass increase during pregnancy [kg]	11.7±7.1	11.3±6.7	12.9±8.4	0.42	0.68	0.55
BMI before pregnancy [kg/m^2]	26.4±5.7	27.1±6.4	25.1±6.0	0.57	0.42	0.33
BMI at birth [kg/m^2]	30.7±5.3	31.2±6.0	29.7±6.7	0.71	0.43	0.43
BMI increase during pregnancy [kg/m^2]	4.3±2.6	4.1±2.5	4.6±3.1	0.30	0.78	0.63
Newborn body mass [g]	3263±554	3297±584	3370±618	0.73	0.64	0.82
APGAR [0-10]	9.2±1.1	9.3±0.9	9.4±1.0	0.90	0.58	0.61

[&] Mann-Whitney U test.

Table S3. Clinical parameters of women with GDM stratified according to *CAPN10* rs2975760 genotype.

Parameters	*CAPN10* rs2975760 Genotype					
	**TT** **n=196**	**TC** **n=67**	**CC** **n=7**	**TT vs TC**	**TT vs CC**	**TC vs CC**
	**Mean ± SD**	**Mean ± SD**	**Mean ± SD**		**p[&]**	
Fasting glucose in an oral glucose tolerance test (mg/dL)	95.8±12.2	96.3±10.8	93.7±8.6	0.32	0.72	0.55
Glucose after 1 h in an oral glucose tolerance test (mg/dL)	155.5±38.5	158.5±33.1	159.7±51.7	0.32	0.82	0.93
Glucose after 2 h in an oral glucose tolerance test (mg/dL)	129.7±36.8	133.7±29.5	120.7±30.5	0.21	0.58	0.28
Daily insulin requirement [unit]	17.1±31.6	19.2±22.4	17.0±21.9	0.06	0.94	0.65
Body mass before pregnancy [kg]	73.6±17.4	72.9±15.9	71.4±16.6	0.93	0.68	0.72
Body mass at birth [kg]	85.4±15.8	84.4±15.2	79.1±13.8	0.68	0.31	0.41
Body mass increase during pregnancy [kg]	11.8±6.9	11.5±7.6	7.8±4.2	0.65	0.08	0.16
BMI before pregnancy [kg/m^2]	26.7±6.0	26.5±5.7	25.7±5.6	0.92	0.71	0.71
BMI at birth [kg/m^2]	31.0±5.6	30.7±5.6	28.5±4.3	0.65	0.23	0.37
BMI increase during pregnancy [kg/m^2]	4.3±2.6	4.2±2.8	2.8±1.5	0.65	0.08	0.17
Newborn body mass [g]	3256±591	3325±502	3416±177	0.74	0.48	0.39
APGAR [0-10]	9.2±1.1	9.4±0.9	10.0±0.0	0.14	0.02	0.04

[&] Mann-Whitney U test.

Table S4. Clinical parameters of women with GDM stratified according to *CAPN10* rs3792267 genotype.

Parameters	*CAPN10* rs3792267 Genotype					
	GG n=132	GA n=108	AA n=30	GG vs GA	GG vs AA	GA vs AA
	Mean ± SD	Mean ± SD	Mean ± SD	p[&]		
Fasting glucose in an oral glucose tolerance test (mg/dL)	96.0±9.1	96.4±15.2	93.3±7.0	0.63	0.13	0.17
Glucose after 1 h in an oral glucose tolerance test (mg/dL)	157.4±36.2	157.7±40.2	146.9±32.5	0.86	0.18	0.21
Glucose after 2 h in an oral glucose tolerance test (mg/dL)	130.4±31.6	133.7±38.3	119.3±34.9	0.91	0.09	0.11
Daily insulin requirement [unit]	17.2±21.5	20.2±38.8	10.0±14.8	0.67	0.08	0.15
Body mass before pregnancy [kg]	74.3±18.1	71.8±15.9	75.1±15.6	0.33	0.53	0.20
Body mass at birth [kg]	85.5±16.7	83.5±14.3	88.0±15.0	0.44	0.33	0.08
Body mass increase during pregnancy [kg]	11.2±7.0	11.8±7.1	12.9±7.2	0.17	0.20	0.90
BMI before pregnancy [kg/m^2]	26.9±6.2	26.3±5.7	26.5±5.8	0.61	0.99	0.81
BMI at birth [kg/m^2]	31.0±5.8	30.7±5.2	31.0±5.4	0.94	0.87	0.70
BMI increase during pregnancy [kg/m^2]	4.1±2.6	4.3±2.6	4.5±2.4	0.16	0.28	0.94
Newborn body mass [g]	3314±562	3201±515	3393±705	0.04	0.15	0.01
APGAR [0-10]	9.3±0.9	9.2±1.0	9.0±1.6	0.70	0.57	0.73

[&] Mann-Whitney U test.

Table S5. Clinical parameters of women with GDM stratified according to *JAZF1* rs864745 genotype.

Parameters	*JAZF1* rs864745 Genotype					
	TT n=79	CT n=136	CC n=55	TT vs CT	TT vs CC	CT vs CC
	Mean ± SD	Mean ± SD	Mean ± SD	p[&]		
Fasting glucose in an oral glucose tolerance test (mg/dL)	96.3±13.6	95.9±11.9	95.2±8.4	0.43	0.49	0.98
Glucose after 1 h in an oral glucose tolerance test (mg/dL)	157.5±39.1	157.8±36.7	151.4±37.2	0.91	0.54	0.48
Glucose after 2 h in an oral glucose tolerance test (mg/dL)	128.5±34.0	133.4±34.6	126.2±37.0	0.32	0.72	0.24
Daily insulin requirement [unit]	16.3±21.1	20.3±35.5	12.7±20.7	0.95	0.15	0.16
Body mass before pregnancy [kg]	72.8±18.3	73.6±16.6	73.7±15.9	0.60	0.59	0.94
Body mass at birth [kg]	85.2±17.9	84.8±15.4	85.3±12.7	0.81	0.80	0.43
Body mass increase during pregnancy [kg]	12.3±7.2	11.2±6.1	11.5±8.9	0.33	0.81	0.60
BMI before pregnancy [kg/m^2]	26.5±6.3	26.6±5.8	26.8±5.9	0.74	0.77	0.90
BMI at birth [kg/m^2]	31.1±6.2	30.7±5.4	31.0±4.9	0.66	0.97	0.50
BMI increase during pregnancy [kg/m^2]	4.5±2.7	4.1±2.2	4.2±3.2	0.29	0.82	0.67
Newborn body mass [g]	3279±526	3244±627	3359±435	0.71	0.64	0.27
APGAR [0-10]	9.4±1.0	9.2±1.2	9.2±0.9	0.14	0.09	0.81

[&] Mann-Whitney U test.
